# Sex Differences in the Relationship between New York Heart Association Functional Classification and Survival in Cardiovascular Disease Patients: A Mediation Analysis of Exercise Capacity with Regular Care Data

**DOI:** 10.31083/j.rcm2308278

**Published:** 2022-08-10

**Authors:** Klaske R. Siegersma, Niels A. Stens, Floor Groepenhoff, Yolande Appelman, Igor I. Tulevski, Leonard Hofstra, Hester M. den Ruijter, G. Aernout Somsen, N. Charlotte Onland-Moret

**Affiliations:** ^1^Laboratory of Experimental Cardiology, University Medical Center Utrecht, Utrecht University, 3508 GA Utrecht, The Netherlands; ^2^Department of Cardiology, Amsterdam University Medical Centers, location VU University, 1081 HV Amsterdam, The Netherlands; ^3^Department of Cardiology, Radboud University Medical Center, 6525 GA Nijmegen, The Netherlands; ^4^Department of Physiology, Radboud University Medical Center, 6525 GA Nijmegen, The Netherlands; ^5^Central Diagnostic Laboratory, University Medical Center Utrecht, Utrecht University, 3508 GA Utrecht, The Netherlands; ^6^Cardiology Centers of the Netherlands, 3584 AA Utrecht, The Netherlands; ^7^Department of Epidemiology, Julius Center for Health Sciences and Primary Care, University Medical Center Utrecht, Utrecht University, 3508 GA Utrecht, The Netherlands

**Keywords:** NYHA classification, mediation analysis, regular care data, survival

## Abstract

**Background:**

The New York Heart Association (NYHA) functional class has extensively been used for risk stratification in patients suspected of heart failure, although its prognostic value differs between sexes and disease entities. Functional exercise capacity might explain the association between NYHA functional class and survival, and can serve as an objective proxy for the subjective nature of the NYHA classification. Therefore, we assessed whether sex-differences in exercise capacity explain the association between NYHA functional class and survival in patients suspected of cardiovascular disease.

**Methods:**

Electronic health record data from 7259 patients with cardiovascular symptoms, a documented NYHA functional class and cardiac stress electrocardiogram (ECG), was analysed. Follow-up for all-cause mortality was obtained through linkage with Statistics Netherlands. A sex-stratified mediation analysis was performed to assess to what extent the proportional heart rate and -workload during ECG stress testing explain the association between NYHA functional class and survival.

**Results:**

In men, increments in NYHA functional class were related to higher all-cause mortality in a dose-response manner (NYHA II vs III/IV: hazard ratio [HR] 1.59 vs 3.64, referenced to NYHA I), whilst in women those classified as NYHA functional class II and III/IV had a similar higher mortality risk (HR 1.49 vs 1.41). Sex-stratified mediation analysis showed that the association between NYHA and survival was mostly explained by proportional workload during stress ECG (men vs women: 22.9%, 95% CI: 18.9%–27.3% vs 40.3%, 95% CI: 28.5%–68.6%) and less so by proportional heart rate (men vs women: 2.5%, 95% CI: 1.3%–4.3% vs 8.0%, 95% CI: 4.1%–18.1%). Post-hoc analysis showed that NYHA classification explained a minor proportion of the association between proportional workload and all-cause mortality (men vs women: 15.1%, 95% CI: 12.0%–18.3% vs 4.4%, 95% CI: 1.5%–7.4%).

**Conclusions:**

This study showed a significant mediation in both sexes on the association between NYHA functional class and all-cause mortality by proportional workload, but the effect explained by NYHA classification on the association between survival and proportional workload is small. This implies that NYHA classification is not a sole representation of a patient’s functional capacity, but might also incude other aspects of the patient’s overall health status.

## 1. Introduction

The New York Heart Association (NYHA) functional classification is widely used to classify the physical limitations of patients with a variety of cardiovascular symptoms related to heart failure. Step-wise increments in the NYHA functional class were related to an increased mortality risk [1], although important sex differences were apparent. In a sex-stratified analysis of data from the Beta-Blocker Evaluation of Survival Trial (BEST), that randomized patients with heart failure, a NYHA class III or IV and reduced left ventricular ejection fraction to either bucindolol or placebo, men with a NYHA class IV had a mortality risk that was almost twice as high compared to NYHA class III. In women with NYHA class IV mortality risk tripled compared to NYHA class III [2]. Registry data from patients with heart failure with reduced ejection fraction showed a similar trend with higher mortality in patients with NYHA classification IV compared to II. In these patients, NYHA class IV was a significant predictor of all-cause mortality in women, but not in men [3]. These results suggest that the NYHA classification measures disease and symptom characteristics differently in men and women.

Although originally designed for patients with heart failure [4, 5], the NYHA classification is now used as a fast and easy tool for risk stratification in a large share of patients visiting a physician with cardiovascular symptoms. We previously showed that NYHA classification also has prognostic value for types of complaints other than complaints associated with heart failure [6]. Nevertheless, the evidence for risk stratification by NYHA classification in cardiovascular complaints other than heart failure remains limited.

Despite its extensive use, NYHA functional class remains a subjective method of cardiovascular disease (CVD) risk stratification [7, 8, 9], as it reflects the physician’s and patient’s judgment of a patient’s physical condition. An aspect of the patient’s physical condition is the ability to initiate and sustain exercise. This ability might explain the powerful prognostic ability of the NYHA classification [7]. Exercise capacity, i.e., the inability to achieve a maximum workload [10, 11, 12, 13] or maximum heart rate during exercise testing [14, 15], is related to an increased risk of cardiovascular disease (CVD) and all-cause mortality in men and women. Moreover, a low exercise capacity was specifically associated with CVD events in women [16]. In general, women present with a lower exercise capacity than men [17, 18]. This may explain the strong prognostic value of the NYHA classification for clinical outcomes in women.

The intermediating effect of variables that represent exercise capacity on the relation between NYHA classification and all-cause mortality might provide us detailed insight in sex differences in the components of the NYHA classification. Therefore, the aim of the present study was to assess sex differences in the extent to which exercise capacity is responsible for the association between NYHA functional class and mortality risk in CVD patients.

## 2. Materials and Methods

### 2.1 Study Population

**Fig. 1.**
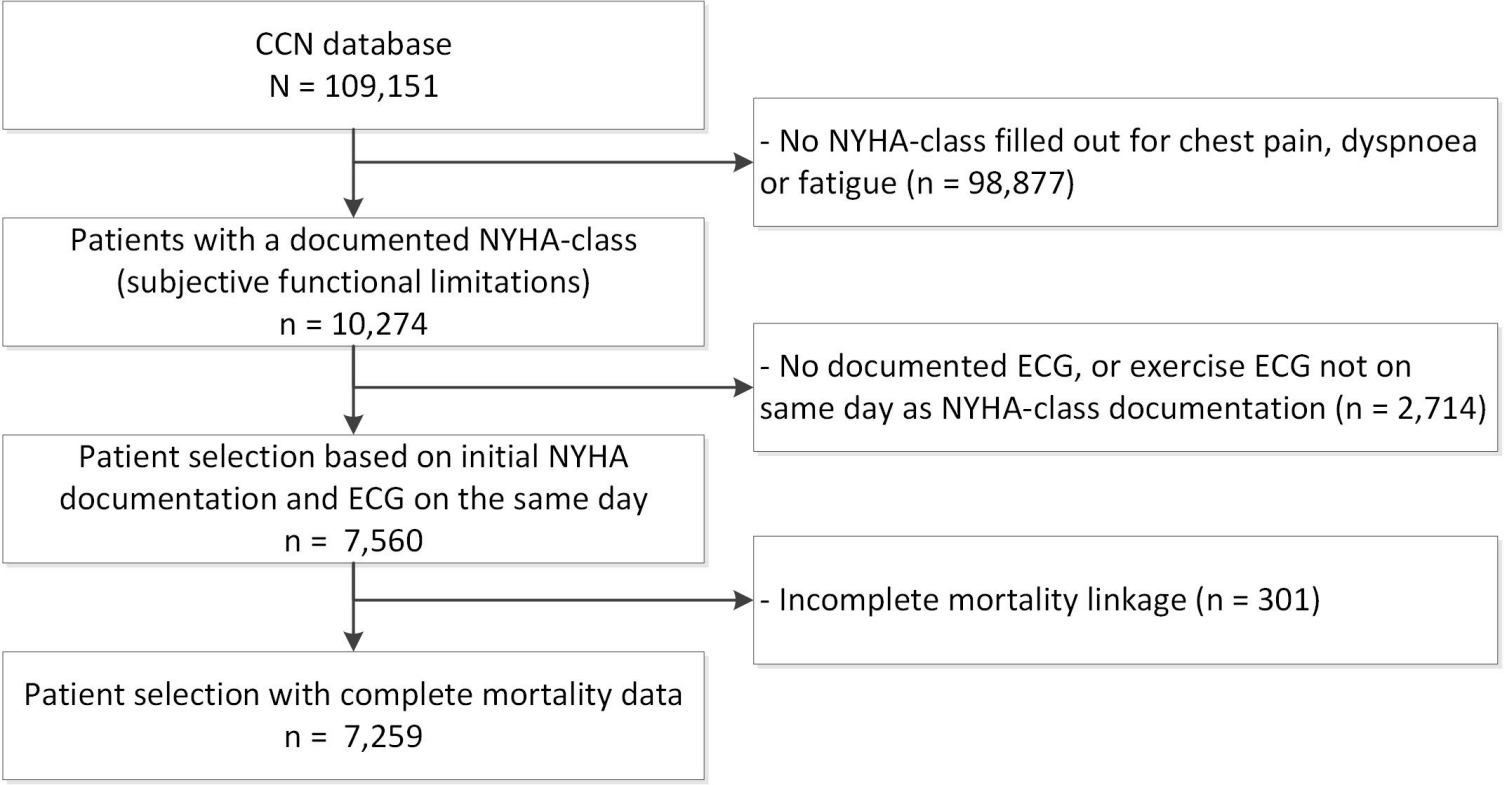
**Flowchart of patient selection**. CCN, Cardiology Centers of the Netherlands; ECG, electrocardiogram; NYHA, New York Heart Association.

Electronic health record data from 2007–2018 of the Cardiology Centers of the Netherlands (CCN) were extracted. The design of the CCN database has been described before [19]. In short, the CCN network contains thirteen “one-stop shop” cardiac outpatient clinics and operates between the general practitioner and hospital cardiologist to facilitate efficient diagnostic cardiac workup. From the available 109,151 patients that were admitted to the CCN between 2007 and 2018, only patients with complete mortality data, the first documented NYHA functional class for dyspnoea, chest pain or fatigue, and stress electrocardiogram (ECG) during the same consult were selected, leaving a final study population of 7259 patients (Fig. 1).

### 2.2 Design

During a consultation, patients received a diagnostic work-up including NYHA functional class for chest pain, dyspnoea or fatigue, a detailed standardized anamnesis by a specialized nurse and cardiologist, where self-reported anthropometrics, symptoms, cardiovascular risk factors, comorbidities and medication use were registered. A NYHA classification of III or IV was converted into a combined class of NYHA class III/IV, as the number of patients documented as class IV was too small to present separately given the privacy regulations of the Statistics Netherlands. Blood pressure measurements (Microlife WatchBP, Microlife AG, Widnau, Switzerland; Medtronic BL-6 Compact, Medtronic, Minneapolis, MN, USA) and a 12-lead ECG (Welch Allyn Cardioperfect recorder, Welch Allyn, New York, NY, USA) were performed both in supine position during rest, and on a watt bike (Lode Corival Eccentric, Lode, Groningen, The Netherlands) during a stress test. Predicted workload during stress was calculated based on the Jones protocol [20] and is dependent on length, age and sex. The corresponding formula is: 3.34* Length -1.43* Age -312-47* Sex , where sex is defined as a logical factor (i.e., women = 1, men = 0). Qualitative text retrieval methods were used to classify the reasons to stop the stress ECG and the conclusion of the stress ECG. The reason to stop was documented as target heart rate achieved, arrhythmia, dyspnoea, chest pain, fatigue, blood pressure and/or painful legs. The conclusion the cardiac stress ECG was documented as either normal, abnormal, inconclusive, incomplete (i.e., target heart rate not reached), myocardial infarction or arrhythmias. The variables used to define exercise capacity were calculated with the following formulas;

(1) $$\begin{array}{c} \text{ Proportional heart rate }=\frac{\text{ Maximum heart rate during exercise }}{\text{ Predicted heart rate during exercise }},\text{ and }\\ \text{ Proportional workload }=\frac{\text{ Maximum workload during exercise }}{\text{ Predicted workload during exercise }} \end{array}$$

Follow-up for all-cause mortality was performed by linkage to Statistics Netherlands (The Hague, Netherlands; i.e., national population registry). Follow-up time was calculated as the interval between age at date of admission to the cardiology center, and age at death [21] or end of follow-up (i.e., February 2020), whichever came first.

### 2.3 Statistical Analysis

Missing values were imputed with sex-stratified multiple imputation using the R package MICE version 3.13.0 [22] with 10 imputations and 50 iterations (**Supplementary Methods 1.1**). To estimate survival function for the different NYHA classes and sexes, a time-to-event analysis using the Kaplan-Meier method and Cox proportional hazards regression was performed. Proportional hazards and linearity were verified using visual inspection of hazard function and residual plots, respectively.

To study the association between NYHA functional class and all-cause mortality, three levels of covariate adjustment were applied. The first level of adjustment was a left-truncated model that inspected the association between NYHA functional class and mortality (age-adjusted model). Secondly, a model was developed with further adjustment for known CVD risk factors and factors associated with mortality (i.e., confounder-adjusted model; **Supplementary Methods 1.2**). To identify factors associated with mortality, NYHA functional class coefficients for mortality were compared between the age-adjusted models with and without the inclusion of the variable of interest. Factors were considered confounders if they affected the NYHA functional class coefficients more than 10%. The third model, the confounder- and intermediate-adjusted model, additionally included the exercise capacity properties as intermediating variables (i.e., proportional workload and proportional heart rate).

To quantify the proportion of the association between NYHA functional class and mortality that could be explained by exercise capacity properties, we used the difference method [23, 24]. Two regression coefficients of the exposure-outcome association were required: the direct effect and the total effect. The direct effect is the coefficient of the NYHA functional class in the confounder- and intermediate-adjusted model, whereas the total effect is the coefficient of the NYHA functional class in the confounder-adjusted model. The proportion of the effect explained by the intermediate (PEE) was subsequently calculated following $\frac{\text{ total effect–direct effect }}{\text{ total effect }}$. NYHA class II and III/IV coefficient estimates were combined via nonlinear transformation to allow the calculation of one PEE per intermediate [25, 26]. Results on the different imputation sets were combined using Rubin’s rules [27]. Bootstrap resampling was used to obtain 95% confidence intervals (CI) around the PEE (**Supplementary Methods 1.3**). Sensitivity analyses were performed per primary NYHA complaint (i.e., fatigue, dyspnoea or chest pain) and for age strata at initial consult (i.e., <65 and ≥65 years).

After evaluation of the first results, a post-hoc analysis was performed that investigated whether NYHA classification and proportional workload accounted for different aspects of risk-stratification in patients with cardiovascular complaints, which was quantified by the PEE of the NYHA functional classification for the association between the proportional workload and mortality. In this analysis, the proportional workload was set as the determinant, whereas the NYHA functional class was added to the association as the intermediating variable. The post-hoc analysis implemented proportional workload as a numerical variable multiplied by 100, converting the proportion into a percentage to ease interpretation.

In all results, NYHA classification was documented with NYHA I as the reference value. All analyses were performed in R (version 4.03, Vienna, Austria) and Rstudio (version 1.3.1093, Boston, USA), pooled according to Rubin’s rules [28], and stratified by sex. An alpha level of 0.05 was considered statistically significant. Data is presented as mean ± standard deviation (SD), median with interquartile range (IQR), or frequency and percentage as appropriate. All linkages and data analyses were performed within the secure environment of Statistics Netherlands, according to Dutch privacy law.

## 3. Results

Patients had a mean age of 58 years, and 52.9% were women. Compared to men, women had overall higher NYHA functional classifications, were older (mean age 58.6 years and 57.2 years for women and men, respectively), had a lower body mass index (BMI, mean BMI 26.6 vs 27.1), and were less likely to have cardiovascular risk factors and comorbidities, e.g., were less often considered smokers (current or former smokers in women: 39.4% and 32.2% vs 40.0% and 38.3% in men), diabetic (7.3% vs 10.0% in, respectively, women and men) and dyslipidaemic (17.3% vs 19.7% in, respectively, women and men). Table 1 (Ref. [29]) gives an overview of these baseline characteristics. Nonetheless, women had a higher median 10-year risk of CVD according to SCORE [29] (3.7 vs 3.0, in women and men, respectively). During both rest and stress, women were more likely to experience dyspnoea, while men were more likely to experience chest pain. Women were able to reach a higher proportional workload despite a similar proportional heart rate compared to males (Table 1). In **Supplementary Table 1**, baseline characteristics are shown stratified by sex and NYHA functional classification.

**Table 1.** **Baseline characteristics of included patients, stratified for sex**.

		Overall	Men	Women	*p*-value
Total patients, n		7259	3419	3840	
Age, years (SD)		57.9 (13.1)	57.2 (13.1)	58.6 (13.0)	<0.001
NYHA functional class, n (%)					0.002
	I	3919 (54.0)	1913 (56.0)	2006 (52.2)	
	II	2908 (40.1)	1297 (37.9)	1611 (42.0)	
	III–IV	432 (6.0)	209 (6.1)	223 (5.8)	
NYHA primary complaint, n (%)					<0.001
	Chest pain	4948 (68.2)	2409 (70.5)	2539 (66.1)	
	Dyspnoea	1575 (21.7)	659 (19.3)	916 (23.9)	
	Fatigue	736 (10.1)	351 (10.3)	385 (10.0)	
Postive family history, n (%)		4874 (67.1)	2149 (62.9)	2725 (71.0)	<0.001
BMI, kg/$m^{2}$ (SD)		26.8 (4.9)	27.1 (4.3)	26.6 (5.4)	<0.001
Smoking status, n (%)					<0.001
	Never	1694 (25.2)	691 (21.7)	1003 (28.3)	
	Former	2364 (35.1)	1222 (38.3)	1142 (32.3)	
	Current	2669 (39.7)	1276 (40.0)	1393 (39.4)	
Diabetes, n (%)		621 (8.6)	340 (10.0)	281 (7.3)	<0.001
Hypertension, n (%)		2621 (36.1)	1230 (36.0)	1391 (36.2)	0.845
Dyslipidemia, n (%)		1333 (18.4)	671 (19.7)	662 (17.3)	0.009
Resting heart rate, beats/min (SD)		73.0 (12.4)	72.1 (12.8)	73.7 (12.0)	<0.001
Arrhythmia during rest, n (%)		71 (1.2)	50 (1.9)	21 (0.7)	<0.001
Medication use, n (%)					
	Antihypertensive use	949 (13.1)	470 (13.7)	479 (12.5)	0.116
	Cholesterol-lowering medication	511 (7.0)	284 (8.3)	227 (5.9)	<0.001
	Anti-diabetic medication	153 (2.1)	88 (2.6)	65 (1.7)	0.012
	Anti-thrombotic medication	459 (6.3)	286 (8.4)	173 (4.5)	<0.001
	Anti-arrhythmic medication	22 (0.3)	<10 (<0.4)	>10 (>0.3)	0.426
	Vitamin K antagonist	66 (0.9)	34 (1.0)	32 (0.8)	0.550
	Other HF medication	10 (0.1)	<10 (<0.4)	<10 (<0.3)	0.256
Left ventricular ejection fraction, n (%)					<0.001
	<40%	58 (0.9)	34 (1.1)	24 (0.7)	
	40–49%	199 (3.1)	135 (4.5)	64 (1.8)	
	≥50%	6221 (96.0)	2826 (94.4)	3395 (97.5)	
HeartSCORE (median [IQR])		3.4 [1.2, 7.8]	3.0 [1.1, 6.9]	3.7 [1.3, 8.8]	<0.001
Nelson-aalen estimator (median [IQR])		0.04 [0.02, 0.06]	0.04 [0.02, 0.06]	0.04 [0.02, 0.06]	0.787
*Stress ECG*					
SBP, mmHg (SD)		199.8 (28.9)	206.1 (28.2)	194.2 (28.4)	<0.001
DBP, mmHg (SD)		86.2 (20.4)	85.6 (20.6)	86.7 (20.3)	0.019
Proportional workload (SD)		0.97 (0.28)	0.86 (0.21)	1.08 (0.29)	<0.001
Proportional heart rate (SD)		1.02 (0.16)	1.02 (0.16)	1.01 (0.16)	0.033
Arrhythmia during exercise, n (%)		2152 (34.9)	1139 (39.4)	1013 (31.0)	<0.001
Reason to stop stress ECG, n (%)					
	Target heart rate reached	1299 (17.9)	641 (18.7)	658 (17.1)	0.079
	Dizziness	223 (3.1)	88 (2.6)	135 (3.5)	0.024
	Fatigue	2794 (38.5)	1217 (35.6)	1577 (41.1)	<0.001
	Chest pain	387 (5.3)	222 (6.5)	165 (4.3)	<0.001
	Painful legs	2261 (31.1)	1,131 (33.1)	1130 (29.4)	0.001
	Arrhythmia	71 (1.0)	48 (1.4)	23 (0.6)	0.001
	Dyspnoea	2341 (32.2)	948 (27.7)	1393 (36.3)	<0.001
	Blood pressure	258 (3.6)	160 (4.7)	98 (2.6)	<0.001
*Follow-up*					
All-cause mortality, n (%)		346 (4.8)	209 (6.1)	137 (3.6)	<0.001
CVD mortality, n (%)		88 (1.2)	53 (1.6)	35 (0.9)	0.018
Follow-up, years [IQR]		5.52 [3.70, 7.59]	5.50 [3.70, 7.61]	5.53 [3.69, 7.58]	0.799

Proportional work load and proportional heart rate are calculated proportions as described in the methods section. BMI, body mass index; CVD, cardiovascular disease; DBP, diastolic blood pressure; ECG, electrocardiogram; IQR, interquartile range; SBP, systolic blood pressure; SD, standard deviation; HeartSCORE, 10 year risk of CVD [29].

**Fig. 2.**
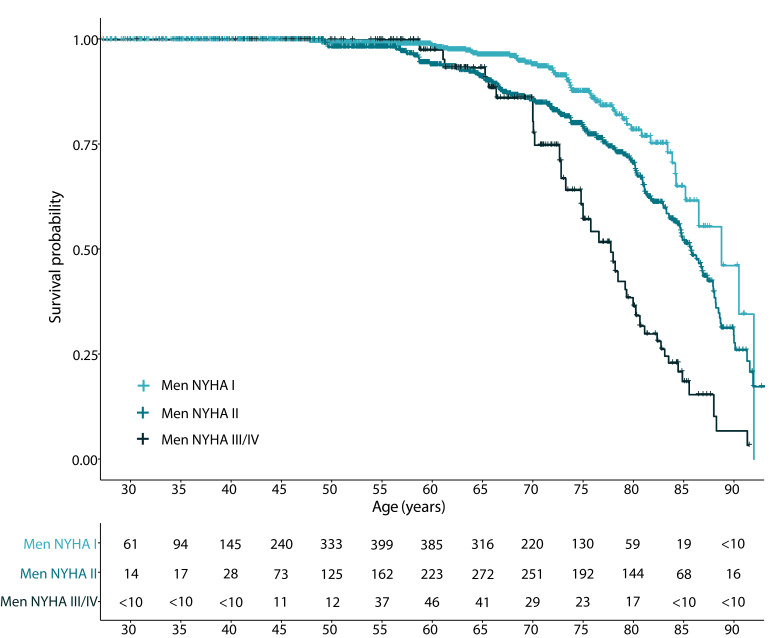
**All-cause mortality during follow-up in men, according to NYHA functional classification**. Following privacy legislation regarding the use of data from Statistics Netherlands, frequencies below 10 in the at-risk table are presented as <10 to prevent patient identification.

**Fig. 3.**
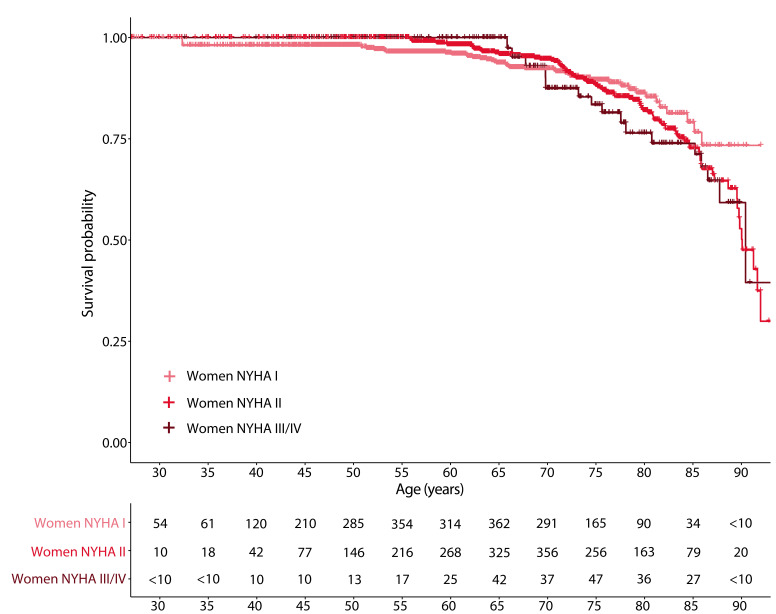
**All-cause mortality during follow-up in women, according to NYHA functional classification**. Following privacy legislation regarding the use of data from Statistics Netherlands, frequencies below 10 in the at-risk table are presented as <10 to prevent patient identification.

During a median follow-up of 5.5 years [IQR 3.70–7.59], 209 men and 137 women died. Survival analysis visualized that increments in NYHA functional class were associated with mortality in both men (Fig. 2 and **Supplementary Fig. 1** using follow-up as the time component) and women (Fig. 3 and **Supplementary Fig. 2** using follow-up as the time component). Univariate analysis showed that BMI (change of NYHA coefficient III/IV in respectively men and women: 9.7% and 15.8%) and conclusion of the ECG stress test (change of NYHA coefficient III/IV in respectively men and women: –7.5% and –25.9%) were confounding factors (**Supplementary Table 2**). These variables were included in the confounding model.

The cox regression analysis confirmed that men classified as NYHA functional class II (HR 1.59, 95% CI: 1.12–2.27) and NYHA functional class III/IV (HR 3.64, 95% CI: 2.31–5.71) had a higher all-cause mortality risk referenced to men classified as NYHA functional class I (Table 2). Similar to men, women classified as NYHA II had a higher all-cause mortality risk than those in class I (HR 1.49, 95% CI: 1.00–2.21) (Table 2). Interestingly, women classified as NYHA functional class III/IV had similar mortality risks to those in class II, when compared to class I (HR 1.41, 95% CI: 0.76–2.62) (Table 2).

**Table 2.** **Univariate and multivariable Cox-regression analysis to evaluate the association between NYHA functional classification and all-cause mortality within men and women with cardiovascular disease**.

Model	n	Events	NYHA II		NYHA III/IV	
			HR (95% CI)	*p*-value	HR (95% CI)	*p*-value
Age-adjusted model men	3419	209	1.62 (1.15–2.29)	0.007	3.92 (2.54–6.06)	<0.001
Age-adjusted model women	3840	137	1.50 (1.01–2.22)	0.045	1.58 (0.86–2.89)	0.141
Confounder model men	3419	209	1.59 (1.12–2.27)	0.011	3.64 (2.31–5.71)	<0.001
Confounder model women	3840	137	1.49 (1.00–2.21)	0.054	1.41 (0.76–2.62)	0.280

Results are presented using NYHA I as the reference level. Variables included in the confounding model were age at presentation to CCN, diabetes, family history, body mass index and the conclusion of the ECG stress test. HR, hazard ratio; CI, confidence interval.

Subsequently, we extended the confounding model by adding potential intermediates for the association between NYHA functional class and all-cause mortality (Table 3). A statistically significant, but small proportion of this association between NYHA and mortality was explained by the proportional heart rate, being more profound in women than in men (men vs women: 2.5% [95% CI: 1.3%–4.3%] vs 8.0% [95% CI: 4.1%–18.1%]). A stronger pattern was observed for the proportional workload (men vs women: 22.9% [95% CI: 18.9%–27.3%] vs 40.3% [95% CI: 28.5%–68.6%]).

**Table 3.** **Results of (1) the mediation analyses of the proportional workload and proportional heart rate on the association between NYHA classification and all-cause mortality, and (2) the post-hoc mediation analysis of NYHA classification on the association between proportional workload and mortality**.

	Event rate (%)	PEE by Proportional workload, % (95% CI)	PEE by Proportional heart rate, % (95% CI)	PEE by NYHA classification, % (95% CI)
Men	6.1	22.9 (18.9–27.3)	2.5 (1.3–4.3)	15.1 (12.1–18.1)
Women	3.6	40.3 (28.5–68.6)	8.0 (4.1–18.1)	4.4 (1.6–7.4)

PEE, proportion effect explained; CI, confidence interval.

The post-hoc analysis showed that lowering in proportional workload was associated with a higher mortality risk (Table 4), in men (HR per % lowering in proportional load of the age-adjusted model: 0.973, 95% CI: 0.966–0.981, HR confounder-adjusted model: 0.974, 95% CI: 0.966–0.981) and to a lesser extent in women (HR age-adjusted model: 0.985, 95% CI: 0.979–0.991, HR confounder-adjusted model: 0.988, 95% CI: 0.982–0.994). The mediation analysis showed that only a minor proportion of the association between proportional workload and mortality was explained by NYHA functional class in both men and women (15.1% [95% CI: 12.1%–18.1%] vs 4.4% [95% CI: 1.6%–7.4%], respectively). **Supplementary Table 2** shows the results of the univariate analysis of confounders.

**Table 4.** **Univariate and multivariable Cox-regression analysis to evaluate the association between the proportional workload and mortality within men and women with cardiovascular disease**.

Model	n	Events	Proportional workload	
			HR (95% CI)	*p*-value
Age-adjusted model men	3419	209	0.973 (0.966–0.981)	<0.001
Age-adjusted model women	3840	137	0.985 (0.979–0.991)	<0.001
Confounder model men	3419	209	0.974 (0.966–0.982)	<0.001
Confounder model women	3840	137	0.988 (0.982–0.994)	<0.001

HR, hazard ratio; CI, confidence interval.

Sensitivity analyses performed to elucidate the influence of age at initial consult and the primary complaint led to similar conclusions. Increments in NYHA functional class were related to all-cause mortality in both men aged <65 and ≥65 years, whilst this trend was absent in women in both age-groups (**Supplementary Table 3**). When stratified by primary complaint, step-wise increases in NYHA functional class were significantly associated with all-cause mortality in men, but not in women (**Supplementary Table 4**).

## 4. Discussion

The aim of the present study was to assess the extent to which exercise capacity properties in men and women separately are responsible for the association between NYHA functional class and all-cause mortality in CVD patients. We first showed that increments in NYHA functional class were related to all-cause mortality risk in both men and women that underwent stress testing, although this seemed to be stronger in men than in women. Second, the proportional workload explained a significant proportion of the association between NYHA functional class and all-cause mortality in men and women, although the majority of this association remained unexplained. Third, the post-hoc analysis showed a lower PEE of NYHA classification in the association between proportional workload and survival compared to the PEE of proportional workload in the association between NYHA classification and survival. Taken together, these results suggest that the NYHA functional class and exercise test provide distinct information within the clinical risk assessment of men and women.

For the current study, we used a unique and large population of patients presenting with a wide variety of symptoms that were admitted to the CCN; an outpatient cardiology clinic which operates between the general practitioner and the hospital. This set-up leads to a population that closely resembles the population with cardiovascular symptoms at the general practitioner’s office. For example, within the current study population, ~53% of the admitted patients were women, providing a solid basis for investigating sex differences within this population. In addition, all centers of the CCN network follow a standardized diagnostic workflow during each consultation, resulting in a high-quality and structured data collection.

The presented study has several limiting factors. First, there are limitations in the selected study population. To enable mediation analysis, only individuals with a documented ECG stress test were selected. This resulted in the exclusion of mainly older women, who suffered from dyspnoea and were classified as NYHA functional class III/IV in whom no ECG stress test was performed. We did not replicate the high prognostic value of the NYHA functional class, especially in NYHA class III/IV, in women that we previously observed [6] whilst sampling from the same population. This suggests that some extent of collider bias was introduced in the presented study by conditioning on the presence of the ECG stress test. This specific selection led to a healthier selected female population with CVD, which distorted the survival estimates in women. In addition, this specific selection prevents accurately estimating the underlying NYHA distribution within this population. Another disadvantage of the selected population is that it also includes patients that have not reached their target heart rate during ECG stress testing. Although these patients have an invalid stress test, exclusion of this population might lead to even more bias as only the very healthy patients were included. Second, although medication use did not differ between men and women, data regarding subsequent treatment was not completely captured. We can therefore not exclude potential sex differences during follow-up, which may have affected all-cause mortality rates in men and women and its relation with NYHA functional class. Third, although all centers of the CCN followed a standardized diagnostic workflow, some cardiologists deviated from the stress ECG protocol due to instability of the patient, which may have influenced estimates of the proportional workload and heart rate and their subsequent PEE. Fourth, only the first-documented NYHA functional class of the patient was selected, which was generally during their initial consult (median consult 1, interquartile range 1:1). However, NYHA functional classification during follow-up may fluctuate in response to disease progression or treatment, which may have affected our hazard ratios in either direction. Fifth, all-cause mortality was considered as the only outcome within this study. Given the majority of risk tools are designed for CVD endpoints, data regarding these endpoints may provide additional insight regarding the domains of the NYHA functional classification. The presented sample size and low number of cardiovascular events hampered performing these analyses. Finally, the retrospective and observational design, despite adopting a multivariate analysis, may also have affected our survival estimates [30].

The NYHA functional class is extensively being used in clinics for a wide variety of applications, including clinical trial inclusion criteria, disease management and prognosis [31, 32]. Previous studies highlighted that increments in NYHA functional class were related to all-cause mortality in both men and women with heart failure with preserved ejection fraction [3], but only in women with reduced ejection fraction [2, 3]. We previously highlighted that increments in NYHA functional class were associated with all-cause mortality in both men and women with CVD [6]. In contrast, the present study, that sampled from the same population [6], showed that stepwise increases in NYHA functional class were related to all-cause mortality risk in men, whilst in women the mortality risk was similar among those classified as NYHA functional class II and III/IV. The introduction of collider bias may therefore have affected the survival estimates of women, although it remains unclear whether this also influenced our PEE estimates obtained in the mediation analysis. We hypothesize that, if these older, excluded women classified as NYHA class III/IV presented with complete stress ECG data, this might have resulted in an overall lower proportional workload in women. Subsequently, a larger proportion of the association between NYHA functional class and all-cause mortality may be explained by the proportional workload in women. Future studies are needed to confirm these hypotheses.

Prior studies have tried to objectify the subjective nature of the NYHA functional class by focusing on exercise [7, 33, 34, 35], and showed that increments in NYHA functional class inversely correlate with objective measures of exercise capacity [33, 34, 35]. Within the present study, the proportional workload explained a significant proportion of the association between NYHA functional class and all-cause mortality in both men and women (22.9% vs 40.3%, respectively), although a large part of this association remained unexplained by variables that represent exercise capacity. In addition, only a minor proportion of the association between the proportional workload and all-cause mortality was explained by NYHA functional class (men vs women: 15.1% vs 4.4%). These results together suggest that the NYHA functional class and ECG stress testing focus on distinct elements within the CVD risk assessment. This has already been hinted at, as previous evidence demonstrated that NYHA functional class poorly differentiated across the spectrum of functional impairment [36, 37, 38]. It may therefore be advised to use an ECG stress test as an extension of the NYHA functional class for clinical risk assessment, rather than as a direct replacement.

Furthermore, large differences in PEE estimates were observed in men and women signifying that the NYHA functional class does not focus on the same disease and symptom characteristics of the risk assessment in men and women. The origin of this discrepancy remains to be elucidated, but we can address the following points. First, differences in presentation of symptoms may prevent uniform classification of NYHA functional class among sexes, as women more often report distinct symptoms [39, 40, 41, 42] and concurrent depressive symptoms [43] compared to men. In addition, sex-discordance between the patient and treating physician may influence symptom perception [44] and risk stratification for clinical outcomes [45, 46, 47, 48]. Unfortunately, we were unable to assess sex-discordances within the present study, which therefore cannot be ruled out. Finally, it seems that women suffer more from functional impairments than men, which is suggested by the larger PEE estimate of the proportional workload in the association between NYHA functional class and all-cause mortality in women. Sex differences in CVD-induced adaptations in cardiac structure [1, 48, 49, 50] may be the cornerstone of these more pronounced functional impairments in women. The differential domains of the NYHA functional class in men and women, paired with its inherent subjective nature, question its reliability within the clinical risk assessment. Nonetheless, the NYHA functional class remains an important prognostic tool for clinical outcomes in both men and women, and cannot directly be replaced by objective variables that represent exercise capacity. This warrants future research to further elaborate on the different domains of the NYHA functional class in men and women.

## 5. Conclusions

This study showed a significant mediation in both sexes on the association between NYHA functional class and all-cause mortality by proportional workload. The effect explained by NYHA classification on the association between survival and proportional workload is small. This implies that the NYHA classification is not a sole representation of the patient’s functional capacity, but extends to the patient’s overall health status. Although the subjective NYHA functional class tends to focus on different domains among sexes, it remains an easy-to-apply and important prognostic tool of CVD risk stratification in both men and women.

## Abbreviations

CCN, Cardiology Centers of the Netherlands; NYHA, New York Heart Association functional classification; ECG, electrocardiogram; CVD, cardiovascular disease; PEE, proportion of the effect explained by the intermediate.

## Supplementary Material

Supplementary material associated with this article can be found, in the online version, at https://doi.org/10.31083/j.rcm2308278.
