# Supplementary material for: Sex Differences in the Relationship between New York Heart Association Functional Classification and Survival in Cardiovascular Disease Patients: A Mediation Analysis of Exercise Capacity with Regular Care Data
Source: Rev Cardiovasc Med. 2022 Aug 10;23(8):278. doi: 10.31083/j.rcm2308278 (PMC11266952; doi:10.31083/j.rcm2308278)
Supplement: Supplementary file 1 [file 2153-8174-23-8-278-s1.docx]

# Supplementary materials

## Multiple imputation

Missing values were imputed using the R package MICE 3.13.0 [1] with 10 imputations and 50 iterations. Multiple imputation was performed for each sex individually to account for effect modification between NYHA functional class and sex. Predictive mean matching was used for continuous variables (i.e. body mass index, systolic and diastolic blood pressure during stress, resting heart rate, proportional heart rate and -workload), logistic regression for binary variables (i.e. presence of diabetes mellitus, dyslipidaemia, positive family history and heart rhythm abnormalities during rest and stress), multinomial logit models for unordered categorical variables with more than 2 levels (i.e. ECG diagnosis: being either normal, abnormal, or inconclusive), and ordered logit models for ordered categorical variables with more than 2 levels (i.e. smoking status). In addition, we included age at initial consult and at event [2], as age represented the underlying time-scale for the Cox proportional hazard regression models; a Nelson-Aalen [3] estimator as well as all-cause mortality as our outcome variable. All predictors were checked for correlation (-0.7 or 0.7), but no significant correlation was observed.

## Univariate analysis

The age-adjusted model was extended with one of the covariates to quantify the relation of the corresponding covariate with all-cause mortality, and was defined as the percentual change of the NYHA coefficients. Factors were considered influential if they changed the NYHA coefficients more than 10% when compared to the age-adjusted model. Although chest pain and dyspnoea during rest and during exercise were considered influential (supplementary table 1), these covariates were not incorporated in the confounding model as the NYHA functional classification is a reflection of these cardiac symptoms. Based on univariate analysis, BMI and conclusion of the stress ECG were selected as relevant confounders (supplementary table 1). These covariates were extended with known CVD risk factors (i.e. diabetes and family history), together constituting the confounding model.

## Bootstrapping

Bootstrapping (1000 bootstrap samples) was performed to compute 95% confidence intervals around the PEE. Bootstrapping was performed per sex and for every imputation set individually. Regression coefficients with and without the intermediate of interest were determined in each imputation set. For each individual bootstrap, obtained total and direct effects of the ten imputation sets were pooled per sex according to Rubin’s Rule. Finally, 1000 PEE’s were obtained of which the 0.025^th^ and 0.975^th^ percentile were taken as the 95% confidence interval around the PEE.

## **Supplementary Table 1. Baseline characteristics of included patients, stratified by sex and NYHA functional classification.**

|  |  | Male | | | Female | | |
| --- | --- | --- | --- | --- | --- | --- | --- |
|  | **Overall** | **NYHA I** | **NYHA II** | **NYHA III/IV** | **NYHA I** | **NYHA II** | **NYHA III/IV** |
| Total patients, n | 7259 | 1913 | 1297 | 209 | 2006 | 1611 | 223 |
| Age, years (SD) | 57.9 (13.1) | 53.32 (12.64) | 62.06 (11.93) | 62.84 (12.11) | 55.30 (12.91) | 61.74 (12.06) | 65.15 (11.88) |
| NYHA primary complaint, n (%) |  |  |  |  |  |  |  |
| Chest pain | 4948 (68.2) | 1575 (82.3) | 730 (56.3) | 104 (49.8) | 1676 (83.5) | 789 (49.0) | 74 (33.2) |
| Dyspnoea | 1575 (21.7) | 182 (9.5) | 398 (30.7) | 79 (37.8) | 182 (9.1) | 619 (38.4) | 115 (51.6) |
| Fatigue | 736 (10.1) | 156 (8.2) | 169 (13.0) | 26 (12.4) | 148 (7.4) | 203 (12.6) | 34 (15.2) |
| Positive family history, n(%) | 4874 (67.1) | 1174 (61.4) | 842 (64.9) | 133 (63.6) | 1381 (68.8) | 1172 (72.7) | 172 (77.1) |
| BMI, kg/m2 (SD) | 26.8 (4.9) | 26.6 (4.2) | 27.5 (4.2) | 28.2 (5.6) | 25.8 (5.0) | 27.4 (5.4) | 28.7 (6.7) |
| Smoking status, n (%) |  |  |  |  |  |  |  |
| Never | 1694 (25.2) | 415 (23.6) | 240 (19.4) | 36 (18.6) | 515 (28.2) | 425 (28.2) | 63 (30.7) |
| Former | 2364 (35.1) | 575 (32.7) | 563 (45.5) | 84 (43.3) | 593 (32.5) | 485 (32.1) | 64 (31.2) |
| Current | 2669 (39.7) | 768 (43.7) | 434 (35.1) | 74 (38.1) | 716 (39.3) | 599 (39.7) | 78 (38.0) |
| Diabetes, n (%) | 621 (8.6) | 125 (6.5) | 181 (14.0) | 34 (16.3) | 96 (4.8) | 156 (9.7) | 29 (13.1) |
| Hypertension, n (%) | 2621 (36.1) | 516 (27.0) | 604 (46.6) | 110 (52.6) | 557 (27.8) | 719 (44.6) | 115 (51.6) |
| Dyslipidemia, n (%) | 1333 (18.4) | 275 (14.4) | 345 (26.7) | 51 (24.4) | 262 (13.1) | 357 (22.2) | 43 (19.5) |
| Resting heart rate, beats/min (SD) | 73.0 (12.4) | 71.5 (12.5) | 72.7 (12.9) | 73.9 (13.3) | 73.3 (11.9) | 74.2 (11.9) | 74.7 (12.9) |
| Arrhythmia during rest, n (%) | 71 (1.2) | <10 (<0.5) | 38 (3.9) | <10 (<4.8) | <10 (<0.5) | 16 (1.3) | <10 (<4.4) |
| Medication use, n (%) |  |  |  |  |  |  |  |
| Antihypertensive use | 949 (13.1) | 168 (8.8) | 261 (20.1) | 41 (19.6) | 168 (8.4) | 278 (17.3) | 33 (14.8) |
| Cholesterol-lowering medication | 511 (7.0) | 92 (4.8) | 174 (13.4) | 18 (8.6) | 72 (3.6) | 140 (8.7) | 15 (6.7) |
| Anti-diabetic medication | 153 (2.1) | 27 (1.4) | 57 (4.4) | <10 (<4.8) | 19 (0.9) | 40 (2.5) | <10 (<4.4) |
| Anti-thrombotic medication | 459 (6.3) | 84 (4.4) | 173 (13.3) | 29 (13.9) | 42 (2.1) | 121 (7.5) | 10 (4.5) |
| Anti-arrhythmic medication | 22 (0.3) |  |  |  |  |  |  |
| Vitamin K antagonist | 66 (0.9) | <10 (<0.5) | 24 (1.9) | <10 (<4.8) | <10 (<0.5) | 28 (1.7) | <10 (<4.4) |
| Other HF medication | 10 (0.1) |  |  |  |  |  |  |
| Left ventricular ejection fraction, n (%) |  |  |  |  |  |  |  |
| <40% | 58 (0.9) | <10 (<0.5) | 25 (2.4) | <10 (<4.8) | <10 (<0.5) | 16 (1.2) | <10 (<4.4) |
| 40-49% | 199 (3.1) | 45 (2.5) | 69 (6.7) | 21 (11.6) | 20 (1.0) | 31 (2.3) | 13 ( 6.2) |
| ≥50% | 6221 (96.0) | 1727 (97.1) | 941 (90.9) | 158 (87.3) | 1893 (98.7) | 1309 (96.5) | 193 (92.3) |
| HeartSCORE (median [IQR]) | 3.4 [1.2, 7.8] | 2.1 [0.7, 4.8] | 4.9 [2.1, 9.4] | 4.5 [2.5, 8.8] | 2.6 [0.9, 6.4] | 5.4 [2.2, 10.5] | 7.2 [2.9, 15.7] |
|  |  |  |  |  |  |  |  |
| *Stress ECG* |  |  |  |  |  |  |  |
| SBP, mmHg (SD) | 199.8 (28.9) | 208.72 (27.30) | 204.76 (28.42) | 191.02 (29.41) | 193.33 (27.78) | 195.84 (28.15) | 190.66 (33.77) |
| DBP, mmHg (SD) | 86.2 (20.4) | 85.74 (21.66) | 85.72 (19.47) | 83.01 (16.67) | 86.00 (20.23) | 87.98 (20.47) | 83.66 (19.46) |
| Proportional workload (SD) | 0.97 (0.28) | 0.91 (0.19) | 0.81 (0.20) | 0.70 (0.22) | 1.11 (0.27) | 1.05 (0.30) | 0.93 (0.34) |
| Proportional heart rate (SD) | 1.02 (0.16) | 1.05 (0.13) | 0.99 (0.18) | 0.94 (0.18) | 1.04 (0.13) | 0.98 (0.17) | 0.94 (0.17) |
| Arrhythmia during exercise, n (%) | 2152 (34.9) | 572 (34.6) | 501 (46.6) | 66 (39.8) | 493 (27.7) | 463 (35.2) | 57 (32.6) |
| Reason to stop stress ECG, n (%) |  |  |  |  |  |  |  |
| Target heart rate reached | 1299 (17.9) | 432 (22.6) | 195 (15.0) | 14 (6.7) | 415 (20.7) | 225 (14.0) | 18 (8.1) |
| Dizziness | 223 (3.1) | 37 (1.9) | 44 (3.4) | <10 | 69 (3.4) | 53 (3.3) | 10 tot 15 |
| Fatigue | 2794 (38.5) | 618 (32.3) | 521 (40.2) | 78 (37.3) | 769 (38.3) | 713 (44.3) | 95 (42.6) |
| Chest pain | 387 (5.3) | 57 (3.0) | 121 (9.3) | 44 (21.1) | 59 (2.9) | 93 (5.8) | 13 (5.8) |
| Painful legs | 2261 (31.1) | 1198 (62.6) | 931 (71.8) | 159 (76.1) | 1356 (67.6) | 1189 (73.8) | 165 (74.0) |
| Arrhythmia | 71 (1.0) | 26 (1.4) | 20 (1.5) | <10 (<4.8) | 16 (0.8) | <10 (<0.6) | <10 (<4.4) |
| Dyspnoea | 2341 (32.2) | 417 (21.8) | 433 (33.4) | 98 (46.9) | 590 (29.4) | 675 (41.9) | 128 (57.4) |
| Blood pressure | 258 (3.6) | 109 (5.7) | 47 (3.6) | <10 (<4.8) | 48 (2.4) | 43 (2.7) | <10 (<4.4) |
|  |  |  |  |  |  |  |  |
| *Follow-up* |  |  |  |  |  |  |  |
| All-cause mortality, n (%) | 346 (4.8) | 49 (2.6) | 123 (9.5) | 37 (17.7) | 39 (1.9) | 82 (5.1) | 16 (7.2) |
| CVD mortality, n (%) | 88 (1.2) | <10 (<0.5) | 36 (2.8) | 11 (5.3) | 10 (0.5) | 22 (1.4) | <10 (<4.4) |
| Follow-up, years [IQR] | 5.52 [3.70, 7.59] | 5.62 [3.99, 7.61] | 5.27 [3.45, 7.69] | 4.74 [2.94, 7.24] | 5.56 [3.87, 7.57] | 5.43 [3.57, 7.63] | 5.28 [3.31, 7.30] |

Proportional work load and proportional heart rate are calculated proportions as described in the methods section. BMI = body mass index, CVD = cardiovascular disease, DBP = diastolic blood pressure, ECG = electrocardiogram, IQR = interquartile range, SBP = systolic blood pressure, SD = standard deviation, HeartSCORE = 10 year risk of CVD.

**Supplementary Table 2. Univariate associations with all-cause mortality.**

| Covariate | NYHA II | | NYHA III/IV | | Proportional workload | |
| --- | --- | --- | --- | --- | --- | --- |
|  | Men | Women | Men | Women | Men | Women |
| Age | REF | REF | REF | REF |  |  |
| BMI | 4.66 | 2.85 | 9.70 | 15.83 | 0,54 | 0,19 |
| Smoking status | -3.43 | -0.19 | -1.26 | 0.97 | -0,70 | 2,15 |
| Positive family history | 0.79 | 0.38 | 1.44 | 2.61 | 0,05 | -0,03 |
| Diabetes | -0.39 | -0.42 | -4.96 | -6.31 | 0,40 | -2,93 |
| Dyslipidemia | -1.79 | -0.62 | 2.44 | -1.08 | -0,17 | 0,29 |
| Hypertension | 0,24 | 4,35 | 0,27 | 2,88 | 0,00 | 0,73 |
| Complaints during rest |  |  |  |  |  |  |
| Chest pain | -12.35 | -6.66 | -37.40 | -54.00 | -5,30 | -7,66 |
| Dyspnoea | -16.28 | -10.94 | -34.00 | -39.76 | -6,65 | -3,88 |
| Fatigue | 3.61 | 2.84 | -3.69 | -8.40 | 3,49 | -1,33 |
| Number of complaints | 0.32 | 0.90 | 2.83 | 3.48 | -0,10 | -0,07 |
| Medication use |  |  |  |  |  |  |
| Antihypertensive use | -2.85 | -1.01 | 0.47 | 0.27 | -0,37 | 0,09 |
| Cholesterol-lowering medication | 2.96 | 0.94 | 3.75 | 1.47 | 0,72 | 1,22 |
| Anti-diabetic medication | 0.53 | -0.18 | -1.48 | -1.93 | 1,17 | -0,53 |
| Anti-thrombotic medication | -1.85 | -1.03 | -0.97 | -0.06 | 0,46 | -0,70 |
| Anti-arrhythmic medication | -1.52 | -0.16 | -0.53 | 0.61 | 0,98 | -0,11 |
| Vitamin K antagonist | -3.68 | 0.57 | 0.73 | -0.51 | -0,30 | 0,06 |
| Other HF medication | -1.71 | -0.26 | 0.76 | -0.01 | -1,80 | 0,27 |
| Resting heart rate | -0.65 | -0.95 | -0.99 | -1.57 | -1,34 | -0,03 |
| Arrhythmia during rest | -4.18 | -1.54 | -4.97 | -7.75 | -1,66 | -2,23 |
|  |  |  |  |  |  |  |
| *Stress ECG* |  |  |  |  |  |  |
| SBP | -3.85 | -7.88 | 0.15 | -1.61 | -10,36 | 2,37 |
| DBP | -0.78 | -0.37 | 4.68 | -1.31 | 0,11 | 0,91 |
| Arrhythmia during stress | 0.15 | -0.05 | -2.15 | 0.41 | -0,15 | 2,32 |
| ECG diagnosis | -6.47 | -4.32 | -7.53 | -25.89 | -3,38 | -17,80 |
| Reason to stop |  |  |  |  |  |  |
| Heart rate | -2.59 | -2.47 | -5.36 | -8.84 | -0,04 | -4,12 |
| Dizzyness | -1.23 | -0.80 | -0.03 | -1.31 | -0,69 | -0,19 |
| Fatigue | -2.68 | -0.24 | -0.41 | 0.50 | 1,05 | 0,16 |
| Chest pain | 3.37 | 3.24 | -1.26 | 0.28 | 0,11 | -0,43 |
| Painful legs | -2.37 | -0.62 | 1.75 | 2.18 | 0,22 | -0,22 |
| Arrhythmia | -0.12 | 0.33 | 0.14 | 0.49 | 0,29 | 0,11 |
| Dyspnoea | -3.88 | -4.90 | -9.81 | -22.68 | -2,26 | 0,20 |
| Blood pressure | -1.79 | -1.43 | 0.08 | -0.98 | -0,54 | -0,31 |

BMI body mass index, ECG electrocardiogram, HF heart failure, HR heart rate, REF reference.

**Supplementary Table 2. Multivariate analysis for men and women with cardiovascular disease, stratified for age at initial consult.**

| Model | N | Events | NYHA II | | NYHA III/IV | |
| --- | --- | --- | --- | --- | --- | --- |
|  |  |  | HR | p-value | HR | p-value |
| Age-adjusted model (<65 years, Men) | 2391 | 47 | 3.00 (1.56, 5.76) | 0.002 | 4.23 (1.62, 11.04) | 0.005 |
| Age-adjusted model (<65 years, Women) | 2509 | 36 | 0.82 (0.41, 1.64) | 0.571 | 1.14 (0.26, 4.87) | 0.865 |
| Age-adjusted model (≥65 years, Men) | 1028 | 162 | 1.29 (0.87, 1.91) | 0.213 | 3.51 (2.15, 5.73) | <0.001 |
| Age-adjusted model (≥65 years, Women) | 1331 | 101 | 2.11 (1.25, 3.57) | 0.006 | 2.09 (1.02, 4.28) | 0.046 |
| Confounding model (<65 years, Men) | 2391 | 47 | 3.30 (1.67, 6.51) | 0.001 | 4.60 (1.69, 12.51) | 0.005 |
| Confounding model (<65 years, Women) | 2509 | 36 | 0.88 (0.43, 1.78) | 0.722 | 1.22 (0.28, 5.40) | 0.795 |
| Confounding model (≥65 years, Men) | 1028 | 162 | 1.26 (0.84, 1.89) | 0.261 | 3.39 (2.05, 5.60) | <0.001 |
| Confounding model (≥65 years, Women) | 1331 | 101 | 2.14 (1.25, 3.64) | 0.006 | 2.07 (0.99, 4.34) | 0.057 |

**Supplementary Table 3. Multivariate analysis for men and women with cardiovascular disease, stratified for primary NYHA complaint.**

| Model | N | Events | NYHA II | | NYHA III/IV | |
| --- | --- | --- | --- | --- | --- | --- |
|  |  |  | HR | p-value | HR | p-value |
| Age-adjusted model (Chest Pain, Men) | 2409 | 114 | 1.47 (0.96, 2.26) | 0.078 | 2.88 (1.51, 5.50) | 0.002 |
| Age-adjusted model (Chest Pain, Women) | 2539 | 59 | 1.36 (0.78, 2.38) | 0.285 | 2.24 (0.84, 5.95) | 0.112 |
| Age-adjusted model (Dyspnoea, Men) | 659 | 78 | 1.50 (0.75, 2.99) | 0.255 | 4.08 (1.88, 8.85) | 0.001 |
| Age-adjusted model (Dyspnoea, Women) | 916 | 60 | 0.86 (0.42, 1.74) | 0.677 | 0.55 (0.20, 1.56) | 0.267 |
| Age-adjusted model (Fatigue, Men) | 351 | 17 | 1.43 (0.29, 7.11) | 0.671 | 4.22 (0.73, 24.47) | 0.132 |
| Age-adjusted model (Fatigue, Women) | 385 | 18 | 1.14 (0.34, 3.81) | 0.830 | 3.27 (0.84, 12.81) | 0.110 |
| Confounding model (Chest Pain, Men) | 2409 | 114 | 1.49 (0.95, 2.32) | 0.084 | 2.94 (1.52, 5.69) | 0.002 |
| Confounding model (Chest Pain, Women) | 2539 | 59 | 1.38 (0.78, 2.45) | 0.272 | 2.20 (0.79, 6.13) | 0.138 |
| Confounding model (Dyspnoea, Men) | 659 | 78 | 1.45 (0.72, 2.93) | 0.303 | 3.72 (1.67, 8.31) | 0.002 |
| Confounding model (Dyspnoea, Women) | 916 | 60 | 0.82 (0.40, 1.68) | 0.594 | 0.49 (0.17, 1.40) | 0.189 |
| Confounding model (Fatigue, Men) | 351 | 17 | 1.36 (0.26, 7.01) | 0.724 | 5.86 (0.94, 36.49) | 0.093 |
| Confounding model (Fatigue, Women) | 385 | 18 | 0.97 (0.27, 3.51) | 0.969 | 2.74 (0.63, 11.95) | 0.213 |

| 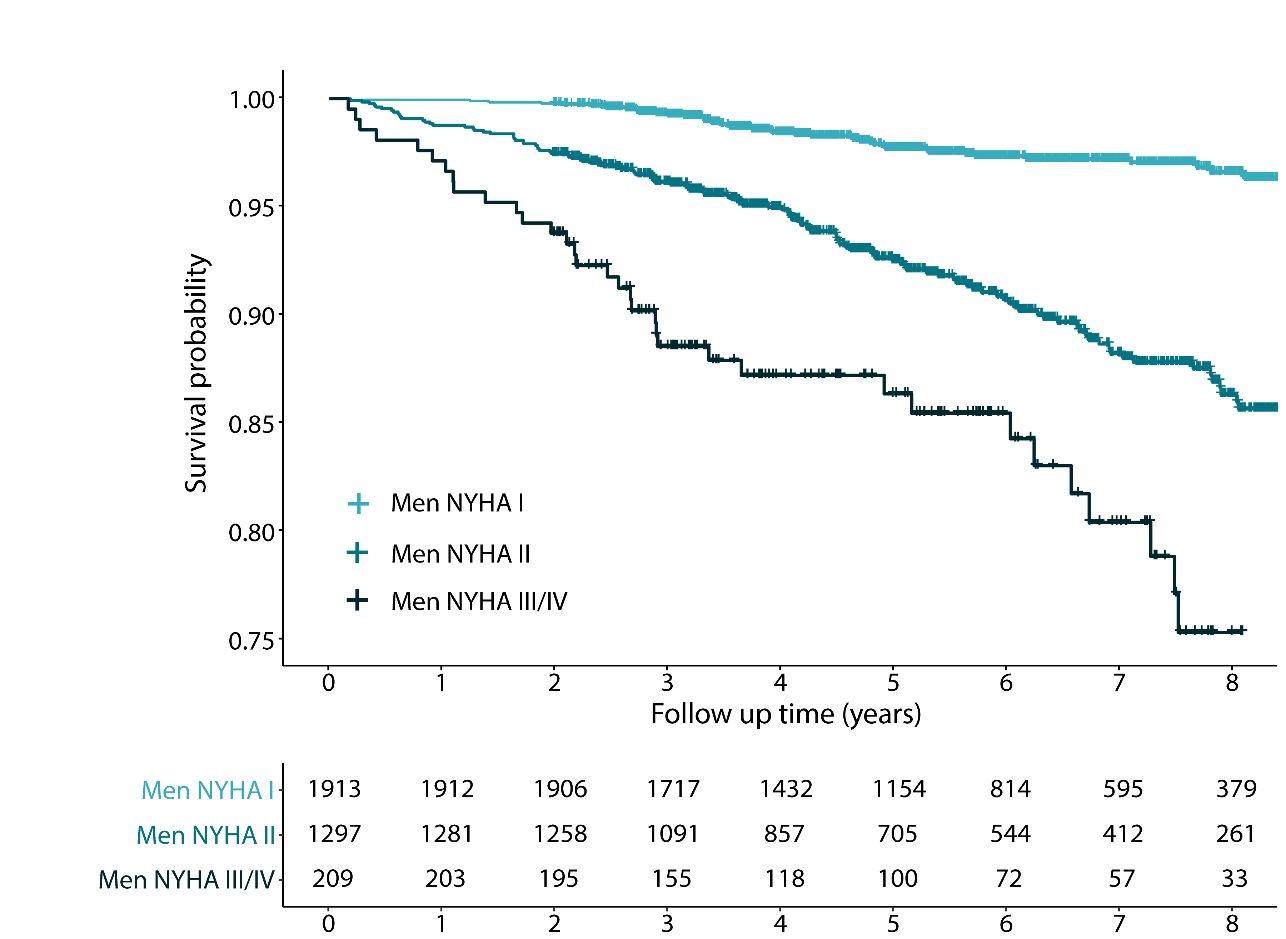 |
| --- |
| **Supplementary Fig. 1. All-cause mortality during follow-up in men, according to NYHA functional classification.** |
| 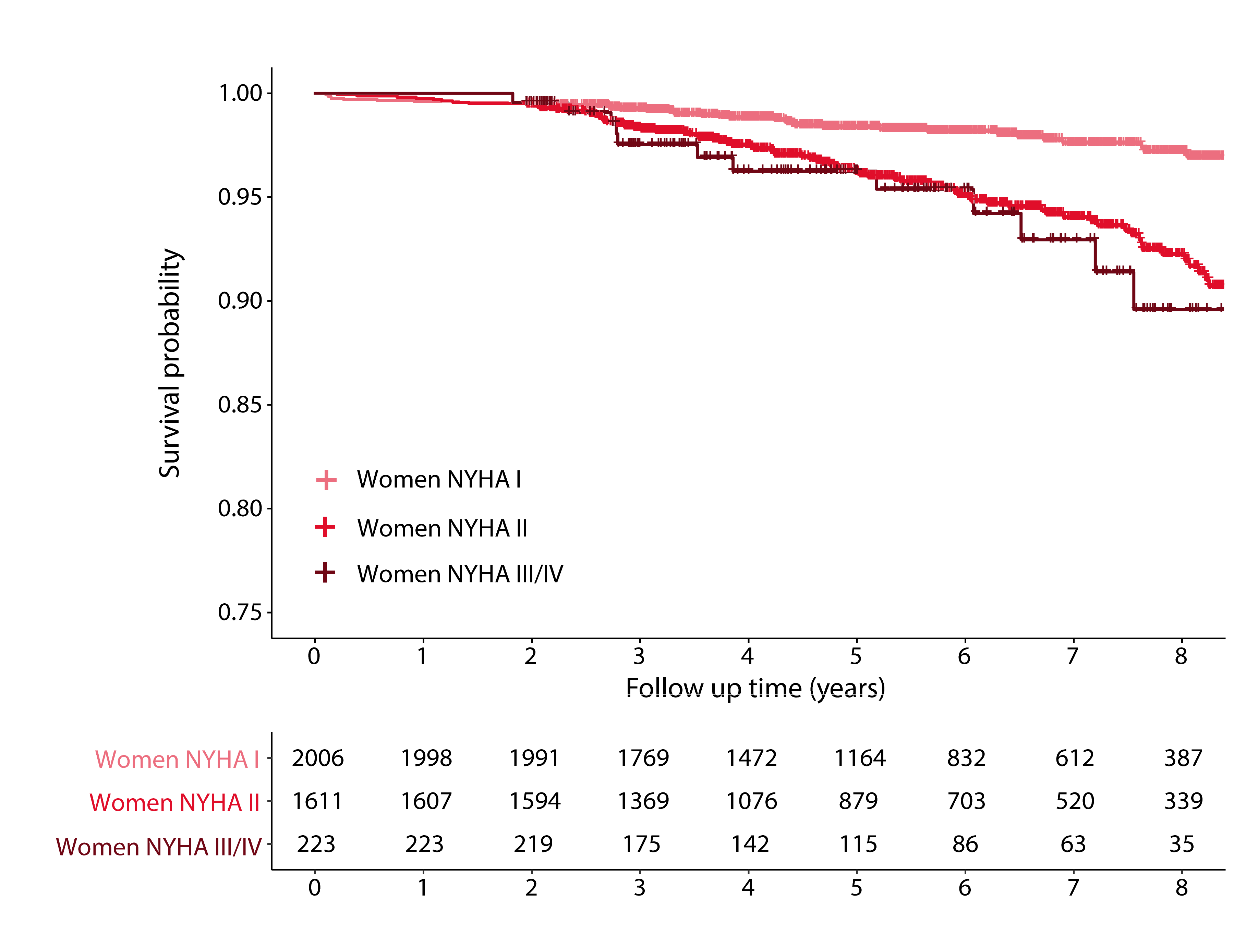 |

**References**

[1] van Buuren S, Groothuis-Oudshoorn K. mice: Multivariate imputation by chained equations in R. Journal of Statistical Software. 2011; 45: 1–67.

[2] Kom EL, Graubard BI, Midthune D. Time-to-Event Analysis of Longitudinal Follow-up of a Survey: Choice of the Time-scale. American Journal of Epidemiology. 1997; 145: 72–80.

[3] White IR, Royston P. Imputing missing covariate values for the Cox model. Statistics in Medicine. 2009; 28: 1982–1998.
